# Efficacy and safety of topical azithromycin therapy in patients with blepharitis and meibomian gland dysfunction

**DOI:** 10.1007/s10384-024-01079-x

**Published:** 2024-07-13

**Authors:** Jun Shimazaki, Gakushi Kito, Mizuka Kamoi, Yoshiyuki Satake

**Affiliations:** 1 Department of Ophthalmology, Tokyo Dental College Ichikawa General Hospital, 5-11-13 Sugano, Ichikawa, Chiba, 272-8513 Japan; 2 Shimazaki Eye Clinic, Tokyo, Japan; 3 Senju pharmaceutical Co., Ltd, Osaka, Japan; 4 Satake Eye Clinic, Chiba, Japan

**Keywords:** Azithromycin, Blepharitis, Bacteria, Meibomian gland dysfunction (MGD), Eyelid

## Abstract

**Purpose:**

To assess the effects of 1% azithromycin ophthalmic solution (AZM) in patients with bacterial blepharitis accompanied by meibomian gland dysfunction (MGD).

**Study design:**

A multicenter, single arm, prospective interventional study.

**Methods:**

AZM was administered to the affected eyes twice daily for the first 2 days and once daily for the subsequent 12 days. Lid margin hyperaemia/redness, collarette at the root of the eyelashes, conjunctival hyperaemia, foreign body sensation, and epiphora were assessed on Days 1, 14, and 28. The Dry Eye-related Quality of Life Score (DEQS) and objectives related to MGD, including lid vascularity, lid margin irregularity, foaming, lid plugging, keratoconjunctival disorders, Marx line, meibum grade, and tear breakup time, were also assessed. Bacterial culture of the conjunctival sac and meibum was performed on Days 1 and 14.

**Results:**

Twenty-four eyes of 24 patients (10 men/14 women, mean age 72.3 ± 13.2) were included. On Days 14 and 28, the total score, lid vascularity, lid plugging, and meibum grade showed significant improvement (*p* < 0.05). On Day 1, 71 strains were isolated from 22 of the 24 eyes (91.7%). *Cutibacterium acnes*, *Corynebacterium* spp., and *Staphylococci* were detected at high frequencies. The overall disappearance rates of the bacteria in the conjunctival sac and meibum at the end of treatment were 65.7% and 58.3%, respectively. No serious ocular or systemic adverse events were observed.

**Conclusion:**

Fourteen-day treatment with AZM was effective in patients with blepharitis accompanied by MGD, and the efficacy of AZM persisted for a period after the treatment.

## Introduction

Blepharitis is characterised by hyperaemia and/or redness of the eyelid margin and is classified as either infectious or non-infectious. The inflammation associated with infectious blepharitis is mainly caused by bacterial infection, and is known to undergo repeated exacerbations and remissions. *Staphylococci*, *Cutibacterium* (formerly known as *Propionibacterium*) *acnes*, and *Corynebacterium* spp. are representative bacteria that cause blepharitis [1, 2]. Lipases produced by these bacteria break down lipids into free fatty acids, which induce inflammation of the eyelid margin [2–4]. Blepharitis can be classified according to the cause and the anatomical site of onset. Inflammation is mainly detected around the orifice of the meibomian gland (MG) in patients with posterior blepharitis. Many patients with posterior blepharitis are also affected by meibomian gland dysfunction (MGD), which is associated with lid vascularity, plugging, and decreased meibum secretion [5]. Hyperkeratinisation of the duct and orifice is thought to be the cause of MGD, and bacterial enzymes, such as lipases, can also obstruct MG orifices by altering the meibum quality and/or decreasing meibum extrusion [6]. Thus, infectious blepharitis with MGD may require comprehensive treatment that includes an anti-bacterial approach [7].

Azithromycin is a 15-membered ring belonging to the macrolide family that inhibits protein synthesis by binding to the 50 S subunit of the 70 S ribosome [8]. A 1% azithromycin ophthalmic drug (AZM; AZIMYCIN® ophthalmic solution 1%; Senju Pharmaceutical Co., Ltd.) was approved in Japan for use in patients with blepharitis in 2019. Although the effects of azithromycin on MGD are reported in several studies [9–11], its effects on patients with concurrent infectious blepharitis and MGD remain unclear. In Japan, the MGD guidelines were published in 2023, and it is expected that the importance of clinical research that evaluates the effectiveness of MGD treatment based on solid definitions will continue to increase [5]. The present study assessed the efficacy of AZM in patients with infectious anterior and/or posterior blepharitis accompanied by MGD via bacteriological studies before and after the administration of AZM.

## Patients and methods

### Study design and ethics

The protocol of this open-label, multicenter, single arm interventional study was approved by the Institutional Review Board of Kitamachi Clinic (approval number: SNJ07951), Tokyo, Japan. This study was conducted in two clinics rather than hospitals because blepharitis is more often diagnosed in clinics than in referral centers. This study was conducted in compliance with the Declaration of Helsinki. Written informed consent was obtained from all patients before participation in the study. This study was registered with the University Hospital Medical Information (UMIN000044348).

### Participants

Eyes with presumed bacterial blepharitis accompanied by MGD were included. The inclusion criteria were: age ≥ 20 years at the time of providing informed consent; objective findings of bacterial blepharitis, i.e., “eyelid margin hyperaemia/redness” score of ≥ 1 (Table 1); and diagnosis of obstructive or hypersecretory MGD in the affected eye. Obstructive MGD was diagnosed according to the Japanese MGD diagnostic criteria published in 2010: presence of any ocular symptoms; presence of abnormal findings around the MG orifices, such as vascularity; and detection of obstruction of the MG orifices due to plugging and reduced meibum expression via moderate digital pressure [12].

**Table 1:** Grading scales for the items used to evaluate blepharitis [13]

Objective score
Lid margin hyperaemia/redness 0 = None 0.5 = Few findings 1 = Slight hyperaemia/redness of the lid margin with no redness of the eyelid skin 2 = Severe hyperaemia/redness of the lid margin with no redness of the eyelid skin 3 = Erosion of the lid margin or redness of the eyelid skin
Collarette at the root of the eyelashes 0 = None 0.5 = Few collarettes 1 = Collarettes at the root of several eyelashes 2 = Collarettes at the root of many eyelashes 3 = Bundled eyelashes due to hypersecretion
Conjunctival hyperaemia 0 = None 0.5 = Few findings 1 = Slight or partial hyperaemia in the conjunctiva 2 = Moderate hyperaemia in the conjunctiva 3 = Severe hyperaemia in the conjunctiva
Subjective score
Foreign body sensation 0 = None 0.5 = Almost none 1 = Grittiness experienced at times 2 = Grittiness experienced but the eyelid can be opened 3 = Grittiness experienced at all times and the eyelid cannot be opened
Epiphora 0 = None 0.5 = Almost none 1 = Eyes moist with tears 2 = Epiphora requiring dabbing at times 3 = Epiphora requiring dabbing frequently

The exclusion criteria were: non-bacterial eye infections; corneal epithelial defects or corneal erosion; systemic or topical administration of other antibiotics and synthetic antibacterials, anti-inflammatory drugs, vasoconstrictors, antihistamines, antiallergic drugs, anti-inflammatory enzymes, or herbal medicines during the study period; the use of warm compresses; and requirement for lid hygiene. In addition, patients scheduled to change their dry eye medication during the study period and those with a history of eye surgery within the preceding 90 days were also excluded from this study.

The affected eye was selected as the target eye in the patients with unilateral involvement, whereas the eye with the highest objective score for blepharitis was selected as the target eye in the patients with bilateral involvement.

### Treatments

All patients were prescribed AZM (Senju Pharmaceutical Co., Ltd.,), administered twice daily for the first 2 days and once daily for the subsequent 12 days. The treatment was initiated on the day of obtaining written informed consent (Day 1). The patients visited the facility every 2 weeks until day 28, and the symptoms and objective data were registered at each visit by the investigators.

### Clinical examination

Ophthalmological examinations were performed at each visit to assess the severity of blepharitis and MGD, and the investigators recorded the scores according to the grading scale (Tables 1 and 2) [13–16]. Fluorescein staining was performed to determine the tear break-up time (TBUT) and identify corneal and conjunctival disorders. A blue-free filter was used in the fluorescein test. TBUT was measured thrice, and the average value was used for the assessment. The cornea was divided into three sections (upper, middle, and lower), and the conjunctiva was divided into two sections (temporal and nasal) for the assessment of corneal/conjunctival punctate epithelial staining. Severity in each section was scored according to the following grading scale: score 0, no disorder; score 1, partial disorder; score 2, disorder affecting > 50% of the area; and score 3, diffused disorder. Sums of the staining scores were used for the assessment.

**Table 2:** Grading scales for the items used to evaluate meibomian gland dysfunction

Lid vascularity [14] 0 = None 1 = Redness of the palpebral conjunctiva with no vascularity around the gland orifices 2 = Redness of the palpebral conjunctiva with vascularity around the gland orifices affecting < 50% of the full length of the lid margin 3 = Redness of the palpebral conjunctiva with vascularity around the gland orifices affecting ≥ 50% of the full length of the lid margin
Lid margin irregularity [14] 0 = None 1 = Fewer than three lid margin irregularities with shallow notching 2 = Three or more lid margin irregularities or deep notching
Foaming [14] 0 = None 1 = Mild findings 2 = Severe findings
Lid plugging [14] 0 = None 1 = Plugging of < 3 gland orifices 2 = Plugging of ≥ 3 gland orifices affecting < 50% of the full length of the lid margin 3 = Plugging of ≥ 3 gland orifices affecting ≥ 50% of the full length of the lid margin
Marx line score [15] 0 = Marx line running entirely along the conjunctival side of the gland orifices 1 = Part of the Marx line in contact with the gland orifices 2 = Marx line running through the gland orifices 3 = Marx line running along the eyelid margin on the side of the gland orifices
Meibum grade [16] 0 = Clear meibum is easily expressed 1 = Cloudy meibum is expressed with mild pressure 2 = Cloudy meibum is expressed with more than moderate pressure 3 = Meibum cannot be expressed even with the hard pressure

### Evaluation of symptoms

All participants completed the validated Japanese version of the Dry Eye-related Quality of Life Score (DEQS) questionnaire, which has also been used as assessment measure of MGD symptoms, at each visit [17–19]. The DEQS questionnaire consists of questions regarding six ocular symptoms and nine effects on daily life: foreign body sensation; dry sensation in the eyes; painful or sore eyes; ocular fatigue; heaviness of the eyelids; redness of the eyes; difficulty in opening the eyes; blurring of vision on watching something; sensitivity to bright light; difficulty in reading, watching television, or looking at a computer or cell phone screen; feeling distracted due to ocular symptoms; ocular symptoms affecting work; difficulty in going out due to ocular symptoms; and depressed mood due to ocular symptoms. The frequency was rated on a Likert scale ranging from 0 (not frequent at all) to 4 (very frequent), and the degree was rated using a Likert scale ranging from 1 (not very concerned) to 4 (very concerned).

### Bacterial sample collection

Samples of the conjunctival sac scraping and meibum were collected on Days 1 and 14 for bacterial testing. The conjunctival sac was scraped after administering preservative-free topical anaesthesia (Oxybuprocaine Hydrochloride Minims® Ophthalmic Solution 0.4%, Senju Pharmaceutical Co., Ltd.). Sterile cotton swabs were used to scrap the conjunctival sac. The lower eyelid was then compressed using tweezers under an operating microscope, and the meibum was carefully collected using sterile cotton swabs without tear or sebum contamination. The samples were stored in a transport medium (ANAport, BIKEN) and transported to the Research Foundation for Microbial Diseases of Osaka University.

### Microbiological methods

The bacterial strains were isolated via direct and enrichment culture methods at the Research Institute for Microbial Diseases of Osaka University, Osaka, Japan. Direct culture was performed under aerobic conditions by inoculating the samples in the following agar medium with or without Tween 80: BD BBL™ Trypticase™ soy agar with 5% sheep blood (Becton, Dickinson and Company), BD BBL™ Columbia agar with colistin and nalidixic acid containing 5% sheep blood (Becton Dickinson and Company), BD BBL™ McConkey II agar (Becton Dickinson and Company), or Chocolate HP agar (Kyokuto Pharmaceutical Industrial Co., Ltd.). The inoculated samples were cultured in an atmosphere containing 5% CO_2_ at 35**°**C for 24–48 h. The samples were subsequently inoculated onto BD™ Columbia agar containing 5% sheep blood (Becton Dickinson and Company) under anaerobic conditions and incubated at 35**°**C for seven days. Enrichment culture was performed by culturing the samples on clinical thioglycolate medium (Eiken Chemical Co., Ltd.) at 5**°**C for two weeks. The API® system (BioMérieux Japan Ltd.) was used to identify the bacterial strains. The minimum inhibitory concentration (MIC) of azithromycin was determined using the broth dilution method in accordance with the protocols from the Clinical and Laboratory Standards Institute [20].

### Statistical analysis

The primary efficacy endpoint was defined as the changes in the total subjective and objective scores for blepharitis from baseline on Day 14. Ten participants per institution were expected to be enrolled over a period of 4 months. Thus, the target sample size was set as 30 participants, assuming a dropout rate of 30%.

Patients who had never received AZM and those without primary endpoint data were excluded from the efficacy analysis. The efficacy data are presented as the mean ± standard deviation (SD). The rate of disappearance of the bacteria detected at the first visit was calculated using the formula: (number of patients in whom bacterium A was detected on Day 1 but not on Day 14/total number of patients in whom bacterium A was detected on Day 1) × 100. The disappearance of bacteria identified to the genus level was defined as a 4-fold or higher difference between the pre-administration MIC and that at the end of administration. The Wilcoxon signed-rank test was used to compare the parameters before and after treatment. The changes in each clinical parameter were set as the dependent variable in the univariate linear regression analysis. The baseline scores for lid vascularity, lid plugging, and meibum grade were set as independent variables. Statistical analyses to determine the changes before and after treatment were two-tailed, and the significance of the alpha level was set at < 0.05.

## Results

### Patient profile and pretreatment characteristics

Twenty-four participants, comprising 10 men and 14 women, were enrolled in this study. The mean age of the participants was 72.3 ± 13.2 years. Sixteen right eyes and eight left eyes were selected as the test eyes. The mean scores for lid vascularity, lid plugging, and meibum grade were moderate to high (Table 3).

**Table 3:** Baseline demographic and clinical characteristics

	Participants (*n* = 24)
Age (years), Mean (SD)	72.3 (13.2)
Gender	
Male, *n* (%)	10 (41.7)
Female, *n* (%)	14 (58.3)
Test eye	
Right, *n* (%)	16 (66.7)
Left, *n* (%)	8 (33.3)
Blepharitis symptoms score	
Objective and subjective total score, Mean (SD)	4.6 (1.43)
Objective total score, Mean (SD)	3.3 (1.27)
Subjective total score, Mean (SD)	1.3 (0.83)
DEQS score, Mean (SD)	23.5 (18.90)^a^
Lid vascularity, Mean (SD)	2.0 (0.78)
Lid margin irregularity, Mean (SD)	0.5 (0.51)
Foaming, Mean (SD)	0.3 (0.53)
Lid plugging, Mean (SD)	2.4 (0.78)
Conjunctival fluorescein staining score, Mean (SD)	0.3 (0.68)
Corneal fluorescein staining score, Mean (SD)	0.6 (0.93)
Marx line score, Mean (SD)	1.5 (1.02)
Meibum grade, Mean (SD)	2.0 (0.46)
Tear break-up time (sec), Mean (SD)	4.5 (2.38)^b^

DEQS, Dry Eye related Quality of life Score; SD, standard deviation

^a^ Calculated using data obtained from 22 participants

^b^ Calculated using data obtained from 18 participants

**Fig. 1:**
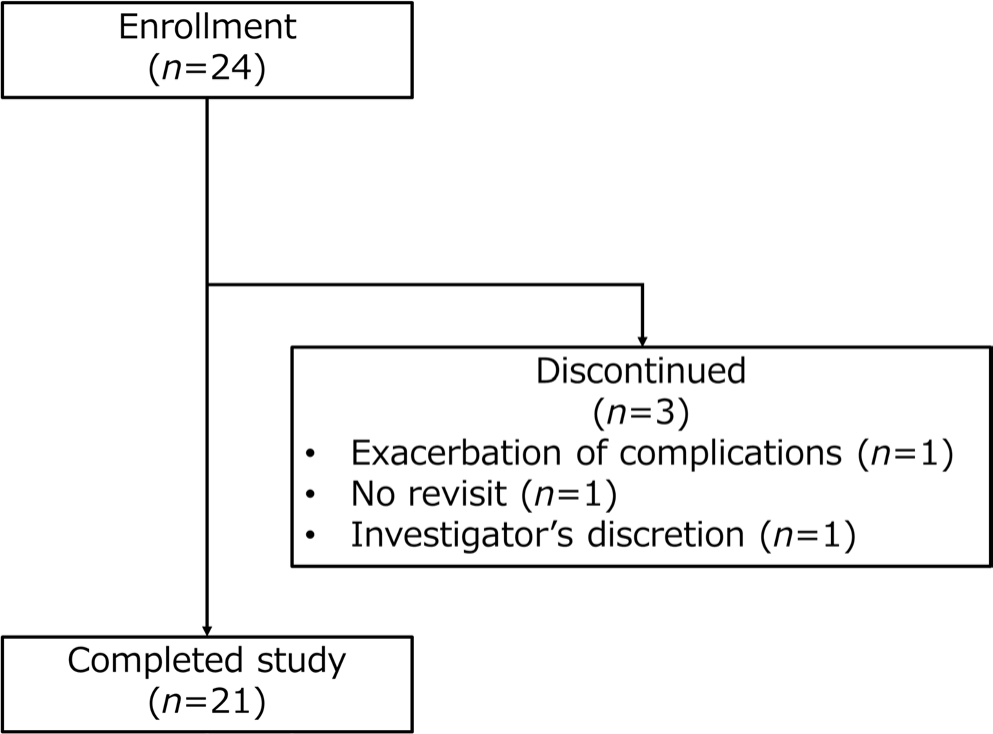
Subject disposition

Three participants (12.5%) were excluded at the investigators’ discretion or due to exacerbation of complications or inability to attend follow-up visits (Fig. 1). Although the participant who was excluded at the investigators’ discretion reported symptoms of eye irritation after the administration of AZM, no objective abnormal findings were observed.

### Symptoms and signs of blepharitis

**Fig. 2:**
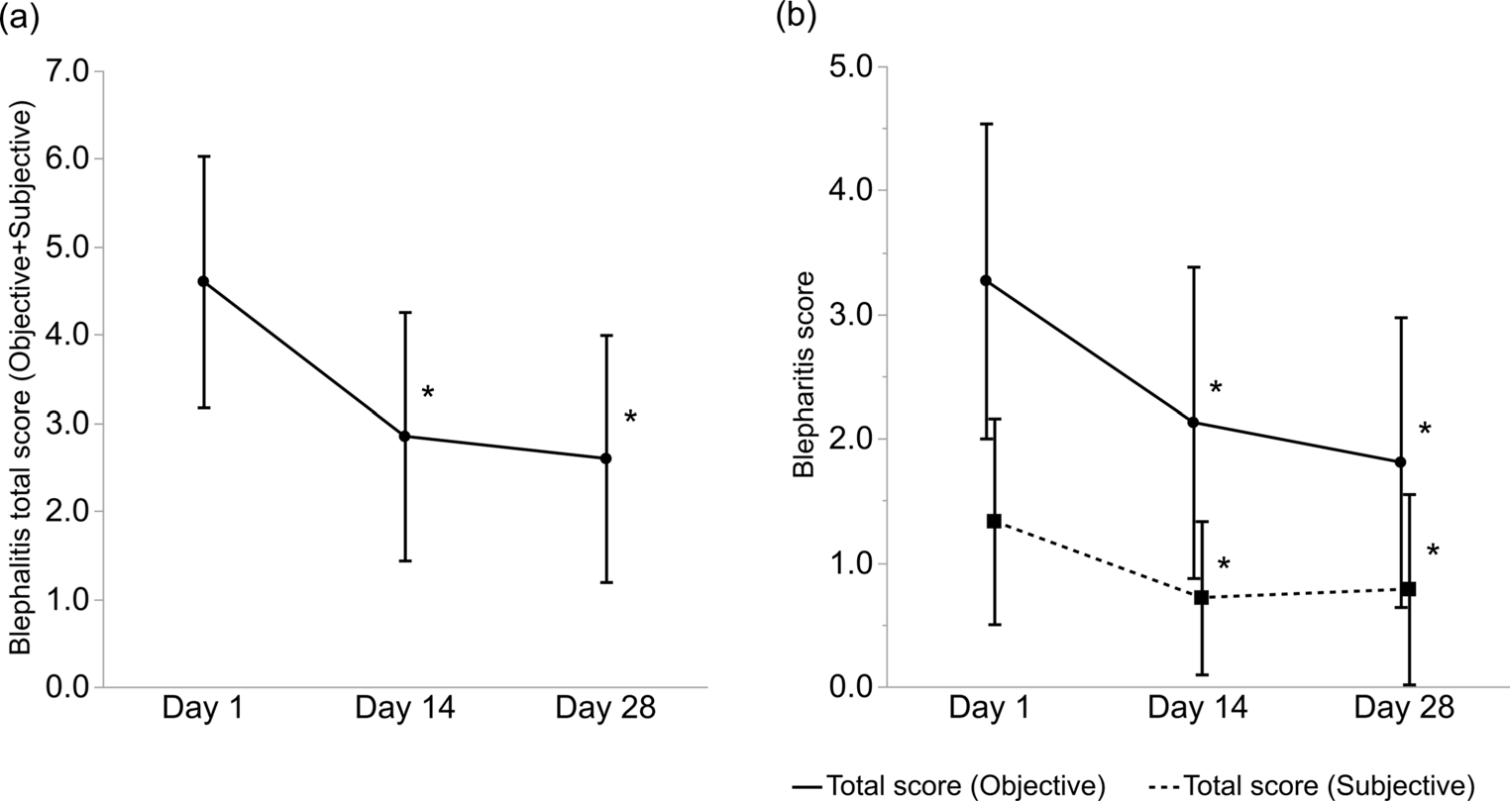
(**a**) Changes in the total scores of blepharitis from baseline. (**b**) Changes in the total objective and subjective scores of blepharitis from baseline. p-values of Days 14 and 28 compared with those at baseline were obtained using the Wilcoxon signed-rank test (* *p* < 0.01). The bars indicate the SD. SD, standard deviation

Figure 2a presents the objective and subjective scores for blepharitis. The scores showed a significant improvement on Day 14 (*p* < 0.001, Wilcoxon signed-rank test), and this improvement was sustained until Day 28 (*p* < 0.001). The total objective and subjective scores for blepharitis were analysed separately (Fig. 2b). The total objective scores showed significant improvement on Days 14 (*p* < 0.001) and 28 (*p* < 0.001). Similarly, the total subjective scores also showed significant improvement on Days 14 (*p* < 0.001) and 28 (*p* = 0.001).

### Signs of meibomian gland dysfunction

**Fig. 3:**
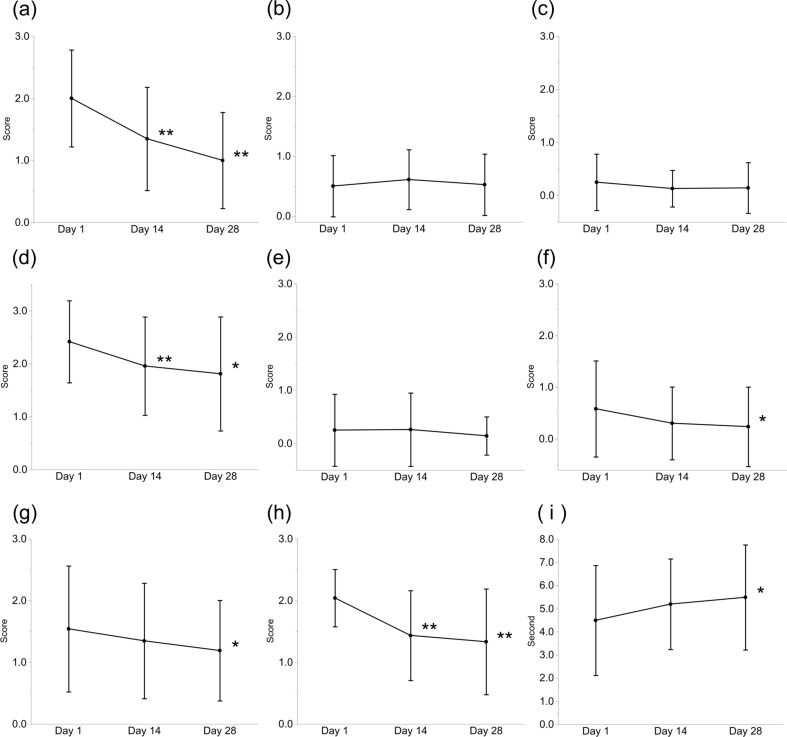
Changes in (**a**) lid vascularity, (**b**) lid margin irregularity, (**c**) foaming, (**d**) lid plugging, (**e**) conjunctival FS score, (**f**) corneal FS score, (**g**) Marx line, (**h**) meibum grade, and (**i**) tear break-up time from baseline p-values of Days 14 and 28 compared with those at baseline were obtained using the Wilcoxon signed-rank test (*: *p* < 0.05, **: *p* < 0.01). The bars indicate the SD. FS, fluorescein staining; SD, standard deviation

Figure 3 shows lid vascularity (panel a), lid plugging (panel d), and meibum grade (panel h). Significant improvements were observed on Days 14 (*p* = 0.002, 0.008, and < 0.001, respectively) and 28 (*p* = 0.001, 0.015, and < 0.001, respectively). Figure 3 shows the corneal fluorescein staining score (panel f), Marx line score (panel g), and TBUT (panel i). Significant improvements in these scores were observed on Day 28 (*p* = 0.029, 0.021, and 0.036, respectively). The remaining findings did not show any significant differences.

### Dry Eye-related quality of life score (DEQS)

**Fig. 4:**
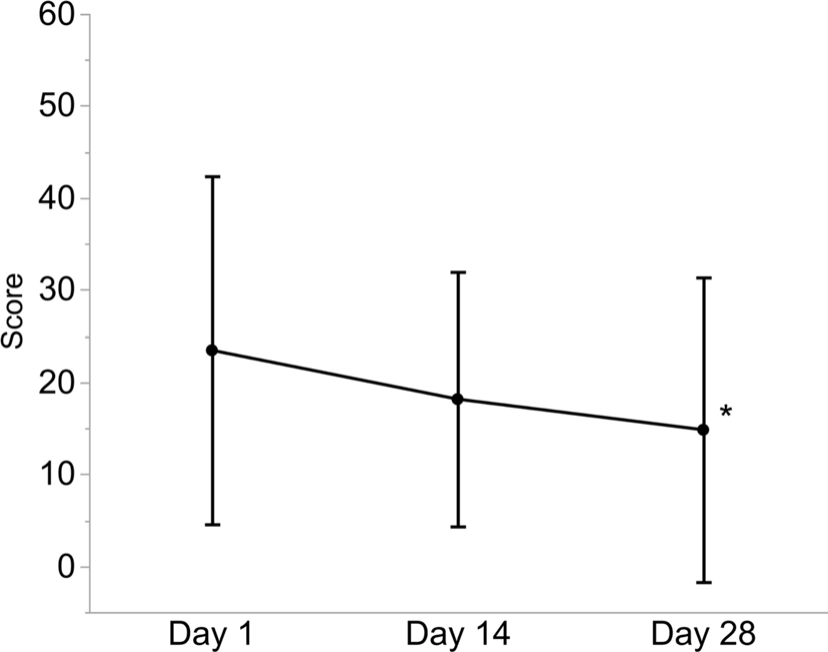
Change in DEQS from baseline. p-values of Days 14 and 28 compared with those at baseline were obtained using the Wilcoxon signed-rank test (*: *p* < 0.01). The bars indicate the SD. DEQS, Dry Eye related Quality of life Score; SD, standard deviation

Figure 4 presents the summary score calculated from the answers obtained from the DEQS questionnaire. The score on Day 14 was lower than on Day 1; however, the difference was not statistically significant (*p* = 0.153). The score showed further improvement on Day 28 even after the discontinuation of the administration of AZM, and a significant difference was observed (*p* = 0.006).

### Factors contributing to the efficacy of AZM

Univariate linear regression analysis was performed to investigate the factors contributing to the therapeutic efficacy of AZM. Seven of the 15 dependent variables contributed to one of the following independent variables: baseline scores of lid vascularity, lid plugging, or meibum grade (Table 4). The seven dependent variables were changes in the value of eyelid margin hyperaemia (*p* = 0.031), lid vascularity (*p* = 0.035), lid plugging (*p* = 0.041), foreign body sensation (*p* = 0.027), conjunctival hyperaemia (*p* = 0.015), conjunctival fluorescein staining score (*p* = 0.022), and corneal fluorescein staining score (*p* = 0.010).

**Table 4:** Univariate linear regression analysis to determine the contribution for improvement of the signs or symptoms after treatment

	Lid vascularity baseline			Lid plugging baseline			Meibum grade baseline		
	B	95% CI	*p*	B	95% CI	*p*	B	95% CI	*p*
Δeyelid margin hyperaemia	-0.29	[-0.53, -0.04]	0.031*	-0.04	[-0.32, 0.23]	0.760	-0.01	[-0.47, 0.44]	0.956
Δeyelash collarettes	0.04	[-0.21, 0.28]	0.774	0.08	[-0.16, 0.33]	0.515	-0.21	[-0.61, 0.18]	0.309
Δconjunctival hyperaemia	-0.04	[-0.21, 0.14]	0.698	-0.01	[-0.19, 0.17]	0.904	-0.35	[-0.61, -0.09]	0.015*
Δforeign body sensation	0.00	[-0.19, 0.19]	> 0.999	-0.21	[-0.38, -0.04]	0.027*	-0.10	[-0.41, 0.22]	0.555
Δepiphora	0.11	[0.00, 0.21]	0.055	-0.08	[-0.19, 0.03]	0.193	-0.18	[-0.35, -0.01]	0.055
ΔDEQS	2.98	[-5.62, 11.57]	0.505	-0.53	[-10.23, 9.18]	0.917	-5.34	[-19.76, 9.09]	0.477
Δlid vascularity	-0.43	[-0.80, -0.06]	0.035*	-0.16	[-0.58, 0.26]	0.467	-0.27	[-0.96, 0.42]	0.448
Δlid margin irregularity	-0.07	[-0.22, 0.08]	0.366	0.09	[-0.06, 0.24]	0.259	-0.02	[-0.28, 0.24]	0.896
Δfoaming	-0.07	[-0.31, 0.17]	0.571	0.01	[-0.24, 0.26]	0.920	0.03	[-0.39, 0.44]	0.902
Δlid plugging	-0.36	[-0.68, -0.04]	0.041*	-0.16	[-0.51, 0.20]	0.403	0.09	[-0.51, 0.68]	0.776
Δconjunctival fluorescein staining score	0.00	[-0.32, 0.32]	> 0.999	-0.15	[-0.47, 0.18]	0.379	-0.61	[-1.08, -0.13]	0.022*
Δcorneal fluorescein staining score	-0.29	[-0.73, 0.16]	0.224	-0.05	[-0.52, 0.42]	0.843	-0.96	[-1.62, -0.29]	0.010*
ΔMarx line score	0.00	[-0.18, 0.18]	> 0.999	-0.14	[-0.31, 0.04]	0.153	-0.18	[-0.48, 0.13]	0.266
Δmeibum grade	-0.07	[-0.38, 0.24]	0.657	-0.11	[-0.43, 0.20]	0.490	-0.08	[-0.60, 0.44]	0.771
Δtear break-up time	-1.16	[-3.01, 0.69]	0.239	-1.26	[-3.01, 0.50]	0.183	-0.83	[-3.53, 1.87]	0.557

Δ, changes in the score between Day 1 and Day 14. DEQS, Dry Eye related Quality of life Score; B, regression coefficient; CI, confidence interval. *: *p* < 0.05

### Microbiological examination

Seventy-one isolates were detected in the samples (22/24 eyes) on Day 1, and 54 isolates were detected in the samples (20/23 eyes) on Day 14 (Table 5). At the initial visit, the positivity rates of the aerobic culture were 70.8% (17/24 eyes) and 66.7% (16/24 eyes) for the conjunctival sac and meibum samples, respectively. The positivity rate of the anaerobic culture was 45.8% (11/24 eyes) in the conjunctival sac and meibum samples. At the end of treatment, the positivity rates of the aerobic culture were 69.6% (16/23 eyes) and 60.9% (14/23 eyes) for the conjunctival sac and meibum samples, respectively. The positivity rates of the anaerobic culture were 34.8% (8/23 eyes) and 39.1% (9/23 eyes) in the conjunctival sac and meibum samples, respectively. The bacterial isolates detected at high frequencies were *C. acnes*, *Corynebacterium* spp., and *Staphylococci*. The bacterial test was repeated after the administration of AZM, and the disappearance rate of the isolates detected on Day 1 was evaluated. The disappearance rates of *C. acnes*, *Corynebacterium* spp., methicillin-susceptible *Staphylococcus epidermidis* (MSSE), methicillin-resistant *Staphylococcus epidermidis* (MRSE), methicillin-susceptible *Staphylococcus aureus* (MSSA), and other strains in the conjunctival samples were 50.0% (5/10 isolates), 60.0% (6/10 isolates), 66.7% (2/3 isolates), 100.0% (4/4 isolates), 50.0% (1/2 isolates), and 83.3% (5/6 isolates), respectively. The disappearance rates of *C. acnes*, *Corynebacterium* spp., MSSE, MRSE, MSSA, methicillin-susceptible coagulase-negative *Staphylococci* (MSCNS), methicillin-resistant coagulase-negative *Staphylococci* (MRCNS), and other strains in the meibum samples were 36.4% (4/11 isolates), 44.4% (4/9 isolates), 0.0% (0/2 isolates), 100.0% (3/3 isolates), 66.7% (2/3 isolates), 100.0% (4/4 isolates), 100.0% (1/1 isolates), and 100.0% (3/3 isolates), respectively. The overall disappearance rates in the conjunctival sac and meibum samples were 65.7% (23/35 isolates) and 58.3% (21/36 isolates), respectively.

**Table 5:** Bacteria isolated via culture on Day 1 and Day 14

Isolated strain	Conjunctival sac		Meibum	
	Day 1	Day 14	Day 1	Day 14
*Cutibacterium acnes*	10	8	11	9
*Corynebacterium* spp.	10	10	9	8
*Staphylococcus epidermidis*				
MSSE	3	5	2	4
MRSE	4	1	3	2
*Staphylococcus aureus*				
MSSA	2	1	3	1
Other CNS				
MSCNS	0	0	4	0
MRCNS	0	0	1	0
*Streptococcus* spp.	2	1	1	0
*Bacillus* spp.	1	2	0	1
*Enterococcus faecalis*	1	0	0	0
*Propionibacterium granulosum*	0	1	0	0
*Proteus mirabilis*	0	0	1	0
*Serratia marcescens*	1	0	1	0
Anaerobic Gram-positive bacilli	1	0	0	0
Total	35	29	36	25

MSSE, methicillin-susceptible *Staphylococcus epidermidis*; MRSE, methicillin-resistant *Staphylococcus epidermidis*; MSSA, methicillin-susceptible *Staphylococcus aureus*; CNS, coagulase negative Staphylococci; MSCNS, methicillin-susceptible coagulase negative Staphylococci; MRCNS, methicillin-resistant coagulase negative Staphylococci

**Fig. 5:**
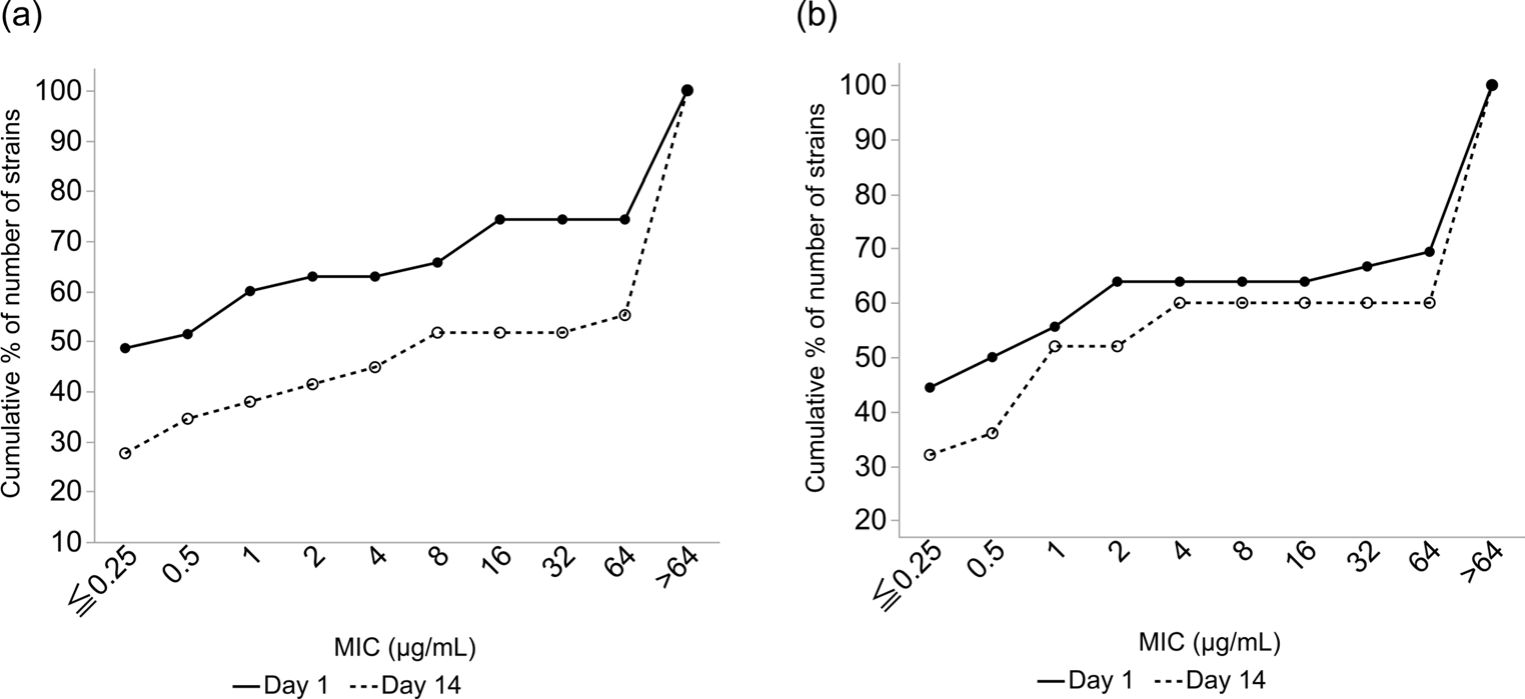
Distribution of the MIC of azithromycin for all strains on Days 1 and 14 shown as a cumulative curve in the (**a**) conjunctival sac and (**b**) meibum. MIC, minimum inhibitory concentration

Figure 5 presents the cumulative curve of MICs for all detected isolates. The proportion of isolates with relatively high MICs against azithromycin in the conjunctival sac and meibum samples increased after administering AZM for 14 days.

### Adverse events

Five ocular adverse events in four patients were reported during the study period (Table 6). The most frequent adverse event was eye irritation, reported in three patients (12.5%). AZM was discontinued due to adverse events in only one patient who experienced exacerbation of eyelid dermatitis. No systemic adverse events were observed.

**Table 6:** Adverse events

	Participants (*n* = 24)
Patients with ≥ 1 adverse events, *n* (%)	4 (16.7)
Eye irritation, *n* (%)	3 (12.5)
Blurred vision, *n* (%)	1 (4.2)
Eyelid dermatitis, *n* (%)	1 (4.2)

## Discussion

In Japan, AZM is indicated for infectious blepharitis, but not for MGD, a precursor lesion of posterior blepharitis [5, 7]. We demonstrated for the first time that 14 days monotherapy with AZM significantly improved both the objective signs and subjective symptoms of patients diagnosed as affected by blepharitis and MGD according to Japanese diagnosis criteria. Additionally, we also assessed the efficacy of AZM by bacteria disappearance rates performing a bacterial culture test. These results are consistent with those of a previous study that assessed the effects of AZM on patients with blepharitis and MGD [9–11, 21–24]. Notably, the effect of AZM was sustained for several weeks after its discontinuation. A previous pharmacokinetic study revealed that azithromycin has a long elimination half-life in the ocular tissue, including the eyelids [25]. The pharmaceutical formulation of AZM contains polycarbophils, which ensures high bioavailability of azithromycin [26]. The pharmacokinetic and pharmaceutical properties of AZM may be contributing to its long-lasting effects.

The therapeutic effects of AZM on blepharitis and MGD may be attributed to several factors. First, azithromycin reportedly promotes lipid secretion by acting on the meibocytes [27]. Increased lipid secretion seems to be important for tear stability. Arita et al. have been demonstrated that AZM plus warm compresses extended TBUT longer than warm compresses alone [11]. Although we did not examine the lipid quantity in the lid margin or tear film, increased lipid secretion may alleviate irritation symptoms and objective signs in MGD.

Second, the anti-inflammatory effects of AZM may be involved. Univariate linear regression analysis in the present study demonstrates that seven dependent variables (changes in the value of eyelid margin hyperaemia, lid vascularity, lid plugging, foreign body sensation, conjunctival hyperaemia, conjunctival fluorescein staining score, and corneal fluorescein staining score) were associated with therapeutic improvements. These variables are related to eyelid inflammation, and previous studies report that azithromycin suppresses inflammatory cytokines, such as IL-1β, IL-8, MMP-9, and TGF-β1 [28]. Therefore, it is likely that the anti-inflammatory effects of AZM are involved in the improvements in eyes affected by blepharitis and MGD observed in the present study.

Third, AZM may have beneficial antibacterial effects of may be related to its effects. The colony counts of *Corynebacterium* spp. and *Staphylococci* are reported to decrease after treatment with 1% azithromycin ophthalmic solution [9, 29]. The results of the present study are consistent with those of these earlier reports. Our results show that the positivity rates of anaerobic strains was 45.8% in both the conjunctival sac and meibum at the initial visit. Zhang et al. report a positivity rate of 10.7% for anaerobic bacteria in both the conjunctival sac and meibum in healthy individuals, whereas the positivity rate for anaerobic bacteria in the conjunctival sac and meibum was as high as 30.8% and 34.3%, respectively, in patients with MGD [30]. *C. acnes* infection is associated with the incidence of blepharitis and MGD [1, 6]. In the present study, MGD was diagnosed based on the presence of ocular symptoms and abnormal findings surrounding the MG orifices. Moreover, all patients enrolled in the present study also had bacterial blepharitis. Therefore, it may be reasonable to assume that the prevalence of anaerobic strains may be higher than that previously reported in patients with MGD [30]. Although the major pathophysiology of hyposecretion in MGD is hyperkeratinisation of the acini, lipolysis by bacterial enzymes, such as lipases, may be related to the alterations in meibum quality. The melting point and viscosity of the abnormal meibum increase, resulting in plugging of the MG orifices [31]. Free fatty acids are also produced via lipolysis, and their decomposition products may induce tissue inflammation and tear film instability [6, 32]. Azithromycin has a broad spectrum of activity; thus, it exerts bacteriostatic effects and inhibits the production of bacterial lipases [33]. Owing to the multiple mechanisms of action, including antibacterial effects, occurring in combination, an improvement in the objective findings of blepharitis and MGD may be observed following AZM treatment.

This study had several limitations. First, it was an open-label study and evaluated the AZM efficacy by the change of each evaluation index between pre- and post-treatment. To clarify more definitive efficacy of AZM monotherapy for treatment blepharitis accompanied by MGD, randomized clinical trials are required. Second, MGD types could not be identified in all participants owing to the lack of a universally accepted definition for hypersecretory MGD. In patients with hypersecretory MGD, subjective symptoms and abnormal findings around the meibomian gland orifices are positive and accelerate the secretion of meibum [34]. Since the diagnostic criteria for hypersecretory MGD are not well-established, analyses were performed using all available data. Therefore, the differences in the effects of AZM on obstructive and hypersecretory diseases could not be studied. Third, the administration of local anaesthetics may have affected the bacterial load. Preservatives are known to inhibit bacterial growth. A preservative-free ophthalmic anaesthetic, 0.4% oxybuprocaine hydrochloride (minims® ophthalmic solution), that is commercially available in Japan was used in the present study. Although its effects on bacterial growth is weak, oxybuprocaine has been suggested to affect bacterial growth [35]. It is possible that the bacterial strains and their disappearance rates after treatment with AZM were affected by the anaesthetic agents used in this study. Lastly, the association between the changes in bacteriological examinations and clinical findings was not clearly demonstrated owing to the limited number of participants. Further large-scale studies are required to clarify this point.

In conclusion, the present study demonstrates that the administration of AZM for 14 days was useful in treating blepharitis in patients with MGD. Moreover, the efficacy of AZM persisted for a certain period after the end of treatment.
